# Exploring immobilization strategies of antimicrobial peptides onto MAO-treated titanium to fight MRSA colonization and preserve osteogenic activity

**DOI:** 10.1016/j.mtbio.2026.102896

**Published:** 2026-02-09

**Authors:** Natália A. Costa, Cláudia Monteiro, Liliana Grenho, Ana R. Ribeiro, Victoria Leiro, Maria H. Fernandes, Paulo N. Lisboa-Filho, M. Cristina L. Martins

**Affiliations:** a UNESP - Universidade Estadual Paulista, Faculdade de Ciências, Bauru, SP, 17033-360, Brazil; b i3S - Instituto de Investigação e Inovação em Saúde, Universidade do Porto, Porto, 4200-135, Portugal; c INEB - Instituto de Engenharia Biomédica, Universidade do Porto, Porto, 4200-135, Portugal; d FEUP - Faculdade de Engenharia, Departamento de Engenharia Mecânica, Universidade do Porto, Porto, 4200-465, Portugal; e BoneLab, Faculdade de Medicina Dentária, Universidade do Porto, Rua Dr. Manuel Pereira da Silva, Porto, 4200-393, Portugal; f LAQV/REQUIMTE, Faculdade de Medicina Dentária, Universidade do Porto, Rua Dr. Manuel Pereira da Silva, Porto, 4200-393, Portugal; g NanoSafety Group, International Iberian Nanotechnology Laboratory, Braga, 4715-330, Portugal; h ICBAS - Instituto de Ciências Biomédicas Abel Salazar, Universidade do Porto, Porto, 4050-313, Portugal

**Keywords:** Titanium, Micro-arc oxidation, Antimicrobial peptides immobilization, Surface functionalization, Methicillin-resistant *Staphylococcus aureus*, Bone-like cells

## Abstract

Alternative therapies to systemic antibiotics are increasingly explored to prevent infections associated with bone implants. Among them, the surface functionalization of titanium with antimicrobial peptides (AMP) is particularly promising due to their broad-spectrum activity and low risk of inducing bacterial resistance. However, a critical challenge remains in achieving both effective antibacterial action and the promotion of osseointegration. This proof-of-concept study investigates different strategies for immobilizing AMP onto bioactive micro-arc oxidation (MAO) coatings on titanium, aiming to combat methicillin-resistant *Staphylococcus aureus* (MRSA) colonization while preserving the osseointegration potential of MAO surfaces. The peptide MSI-78 was immobilized either by physical adsorption or covalent grafting, using 1,1′-carbonyldiimidazole (CDI) coupling agent or poly(ethylene glycol) (PEG) spacer. All immobilization strategies preserved the heterogeneous porous architecture and calcium/phosphorus doping of the complex MAO coatings. Prior to bacterial incubation, the surfaces were pre-conditioned with human plasma proteins. MSI-78, whether by physical adsorption or covalent grafting, killed MRSA after 5 h, but also promoted bacterial adhesion to the surface. In contrast, the combined strategy of grafted PEG and physically adsorbed AMP promoted a remarkable antibacterial effect, by reducing MRSA colonization and killing about 80% of adherent bacteria. Regardless of the immobilization strategy, bacterial killing appeared to occur via contact-mediated membrane disruption. Moreover, these PEGylated MAO surfaces with adsorbed AMP maintained excellent cytocompatibility with bone-like cells and supported osteogenic response, underscoring their potential as bioactive coatings for titanium implants.

## Highlights

• Antimicrobial peptides (AMP) were successfully immobilized onto MAO TiO_2_ surfaces.
• AMP immobilization occurred via physical adsorption or covalent grafting.
• CDI coupling or PEG spacer were used to modulate conjugation.
• AMP immobilization provided surface bactericidal activity against MRSA.
• Grafted PEG + adsorbed AMP enhance antibacterial action and support osseointegration.

## Graphical abstract

**Figure:**
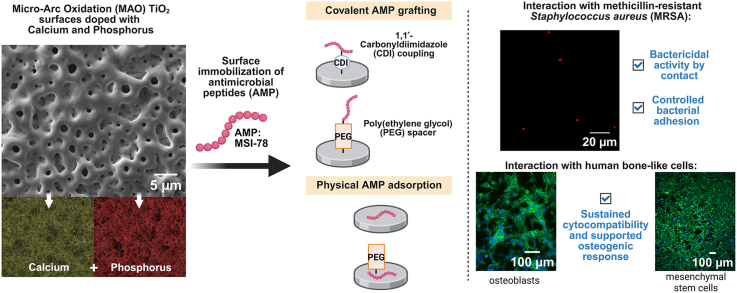

## 1 Introduction

The alarming rise of antibiotic-resistant bacteria has been recognized by the World Health Organization (WHO) as one of the most critical public health threats. This issue significantly compromises the prevention and treatment of infections, including those associated with bone implant surgeries [1,2]. Antimicrobial peptides (AMP) have emerged as promising alternative therapeutic agents, exhibiting broad-spectrum antimicrobial activity and primarily targeting bacterial membranes, which reduces the likelihood of resistance development [[3], [4], [5], [6], [7], [8], [9]]. MSI-78 (commercially known as Pexiganan; sequence: GIGKFLKKAKKFGKAFVKILKK) is a synthetic analog of magainin 2, a natural host defense peptide from the skin of the African frog *Xenopus laevis*. This peptide forms an α-helix arrangement and exerts bactericidal effects via membrane disruption, demonstrating efficacy against over 3000 clinical bacterial isolates, including microorganisms often found at device-related infectious sites, such as the main colonizer of metallic implants, *Staphylococcus aureus*, and its multidrug-resistant variant, methicillin-resistant *S. aureus* (MRSA) [[10], [11], [12]]. Notably, MRSA is listed by the WHO among the 16 antibiotic-resistant bacteria posing the greatest threat to human health [13]. In addition, previous *in vivo* studies reported no toxic responses to MSI-78 [14,15], and clinical trials have confirmed its safety and tolerability in human subjects [16].

Despite their exceptional bactericidal potential, unbound AMP have presented some significant limitations hindering their translation to the clinic. These include AMP cytotoxicity at high concentrations, as well as decrease in AMP activity due to their susceptibility *in vivo* to proteolytic degradation, self-aggregation, and co-precipitation with proteins [5]. One way to circumvent these challenges involves the AMP immobilization onto the biomaterials surface, which enables localized antibacterial action directly at the implant site, where it is most required [5,17,18]. AMP immobilization onto solid substrates can occur through either physical adsorption or covalent grafting. There is no exclusive and ultimate way to perform AMP immobilization; in fact, the process must be tailored based on several parameters. These include peptide type and concentration, substrate, chemical coupling methods, spacer linkers’ presence and properties (*e.g.* chemical nature, length, flexibility), and the AMP's exposure, mobility, and orientation after immobilization. Different routes have been employed to functionalize titanium (Ti) surfaces with AMP for orthopedic and dental applications [3,4,9,19]. Compared to untreated Ti, AMP-conjugated Ti surfaces have demonstrated higher antibacterial efficacy in both *in vitro* [[20], [21], [22], [23], [24], [25], [26], [27], [28], [29], [30]] and *in vivo* [25,28,31,32] studies.

However, antibacterial activity alone may not be enough for the successful integration of implants. An ideal next-generation implant surface should be capable of promoting osseointegration while avoiding bacterial contamination [[33], [34], [35]]. To enhance bone formation, micro-arc oxidation (MAO) has been extensively studied as an effective technique for producing porous TiO_2_ coatings with high adhesion strength, often doped with calcium phosphates on Ti substrates [[36], [37], [38], [39], [40], [41], [42], [43], [44], [45], [46], [47], [48], [49], [50]]. Beyond this, MAO layers have consistently exhibited superior mechanical performance relative to bare Ti, a result derived from their composition of crystalline TiO_2_ structures [51]. These collective attributes have supported the adoption of MAO coatings in commercially available biomedical implants [52]. Although bare Ti surfaces have been widely treated by AMP, the integration of complex MAO coatings with AMP immobilization remains insufficiently investigated, as highlighted in recent reviews [53]. To date, only one study has examined a MAO surface post-treated with AMP, applying multilayers of polydopamine, peptide LL-37, and the phospholipid POPC [54], with a focus on controlled AMP release.

**Scheme 1:**
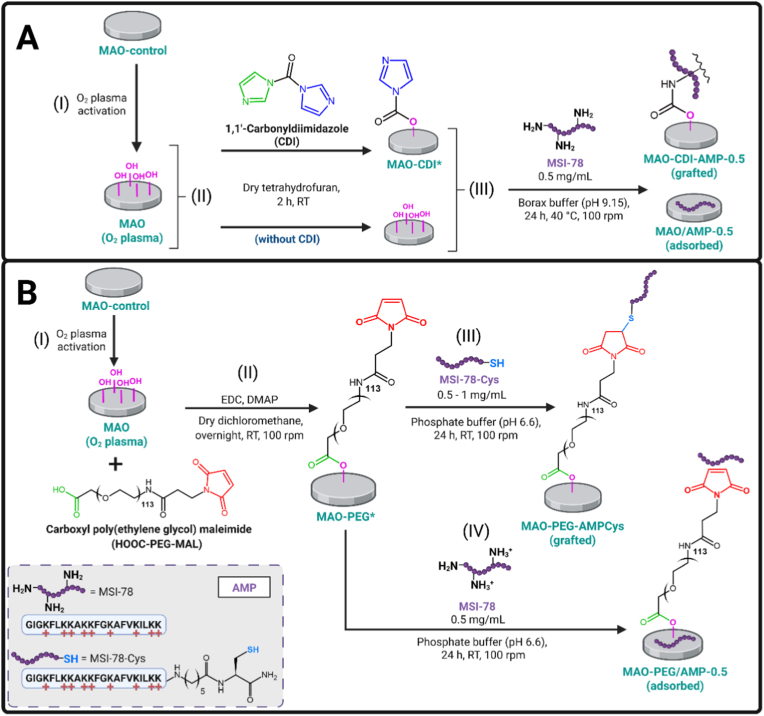
Representation of the strategies of AMP immobilization onto MAO surfaces (not to scale). First, MAO-control coatings were activated with hydroxyl (-OH) groups by O_2_ plasma (I), to be further linked with the peptides by (A) CDI coupling agent or (B) HOOC-PEG-maleimide spacer. Amines (-NH_2_) from the peptide MSI-78 react with CDI (A-III). The cysteine (Cys) from the peptide MSI-78-Cys covalently binds to the maleimide (MAL) group from the PEG spacer (B-III). In parallel, surfaces with physical AMP adsorption were also produced (A-III and B-IV). Controls are MAO-CDI∗ and MAO-PEG∗ immersed in buffers without AMP (MAO-CDI and MAO-PEG samples, respectively). CDI: 1,1′-carbonyldiimidazole; PEG: poly(ethylene glycol); EDC: 1-(3-dimethylaminopropyl)-3-ethylcarbodiimide hydrochloride; DMAP: 4-(dimethylamino)pyridine; RT: room temperature.

Thus, this proof-of-concept work aims to explore MSI-78 immobilization strategies, namely physical adsorption and covalent grafting, onto MAO Ti surfaces. For AMP grafting, two different approaches were investigated (Scheme 1). The 1,1′-carbonyldiimidazole (CDI) coupling agent allowed a more direct AMP conjugation to the surface, with a non-specific (random) reaction (via free amines of the peptide). In the other approach, AMP was grafted using a long chain spacer of poly(ethylene glycol) (PEG), with a selective reaction (thiol-maleimide coupling). These chemistries were chosen for modulating peptide's orientation, exposure, and mobility [20,[55], [56], [57], [58], [59], [60], [61], [62], [63]]. The antibacterial performance against surface-adherent MRSA was evaluated under conditions simulating physiological environments by pre-incubating the surfaces in human plasma. The most promising AMP-functionalized MAO coatings were then assessed for their effects on bone-like cells viability, adhesion, proliferation, and osteogenic behavior.

## 2 Materials and methods

### 2.1 Micro-arc oxidation

Grade 4 Ti substrates (disks of 12.6 mm diameter; Acnis Brazil) were functionalized by MAO, according to previous work [64]. Firstly, the surface of the disks was ground using silicon carbide (SiC) sandpapers with 150 and 800 mesh, and then ultrasonically cleaned in 2-propanol for 10 min. The following chemical etching was done in acidic solution for 5 s, using the same volume proportion of Type II water (purified water with resistivity of >1 MΩ cm, a conductivity of <1 μS/cm and <50 ppb of total organic carbons), hydrogen fluoride (HF) and nitric acid (HNO_3_) (Honeywell, Fluka). Samples were then cleaned in 2-propanol (10 min) and Type II water (5 min) in an ultrasound bath, and dried at room temperature (RT). Afterwards, the MAO process was carried out using a two-electrode electrochemical cell set-up, in which the samples and a grade 2 Ti plate (Sandinox) served as working (anode) and counter (cathode) electrodes, respectively. The anode and cathode were placed 7 cm apart and connected to a direct current (DC) power supply (Keysight Technologies N5772A). A potential difference of 300 V and a limiting current of 2.5 A were applied. The electrolyte solution for anodization was prepared in Type II water, with 0.35 M calcium acetate monohydrate and 0.02 M β-glycerophosphate disodium salt pentahydrate (reagents from Sigma-Aldrich), in order to incorporate calcium and phosphorus as bioactive agents. The electrochemical treatment of each sample lasted 1 min, under magnetic agitation (500 rpm) at RT. After that, samples (designated MAO-control) were rinsed with Type II water and 2-propanol, and dried with air stream at RT.

### 2.2 O_2_ plasma activation

MAO-control samples were exposed to O_2_ plasma to activate their surface with hydroxyl (-OH) groups [64]. Samples were first vacuum-dried (20-40 mbar) at 100 °C for 24 h, to minimize surface physically adsorbed water. After the drying stage, the heating was turned off, and the samples were maintained under vacuum for 2 h. Subsequently, the samples were transferred to the plasma cleaner equipment (Tergeo, Pie Scientific, TG100). Plasma was generated by using a radio frequency (RF) source at 75 W and pulse of 255 (N/255), and an industrial-grade oxygen working gas at a flow rate of 50 sccm. The O_2_ plasma treatment was performed in direct mode for 2 min at a chamber pressure of 1.3 mbar.

### 2.3 Peptides

Peptides MSI-78 (sequence: GIGKFLKKAKKFGKAFVKILKK) and MSI-78-Cys (sequence: GIGKFLKKAKKFGKAFVKILKK-ahx-Cys) were acquired from GenScript Biotech Corporation (Piscataway, NJ, USA), both with ≥95% purity. The cysteine (Cys) residue was attached to a flexible 6-amino-hexanoic acid (ahx) spacer placed at the C-*terminus* of the original MSI-78 sequence. The purchased lyophilized peptides were dissolved in sterile-filtered 0.1 M acetic acid solution, aliquoted, freeze-dried, and stored under nitrogen at −20 °C until further use.

### 2.4 Surface-peptide reactions

Immediately after plasma activation as described in Section 2.2 (please, see Scheme 1, steps A-I and B-I), the MAO (O_2_ plasma) samples were transferred to a glove chamber with a dry N_2_ atmosphere, and incubated in solutions containing coupling agents or linkers/spacers for conjugation, namely 1,1′-carbonyldiimidazole (CDI) or poly(ethylene glycol) (PEG). All glass materials used in the glove chamber were previously dried (overnight, 80 °C). CDI and PEG solutions were freshly prepared inside the glove chamber.

#### 2.4.1 1,1′-Carbonyldiimidazole (CDI) coupling

The CDI-based conjugation approach is illustrated in Scheme 1A. MAO (O_2_ plasma) samples were reacted with the CDI molecule (Aldrich), by immersion in 30 mg/mL CDI solution prepared in anhydrous tetrahydrofuran (THF; Thermo Scientific) (Scheme 1, step A-II). After 2 h incubation at RT, MAO-CDI∗ samples were rinsed three times with THF, quickly dried with a gentle stream of argon, and immediately immersed in peptide solution for covalent AMP grafting.

MSI-78 was dissolved at 0.5 mg/mL in a fresh and sterile-filtered borax buffer (0.01 M sodium tetraborate decahydrate (Sigma-Aldrich) in Type II water at pH 9.15). MAO-CDI∗ samples were incubated with the AMP solution for 24 h, at 40 °C and 100 rpm in an orbital shaker oven (IKA KS 3000) (Scheme 1, step A-III).

After AMP incubation, MAO-CDI-AMP-0.5 samples (“0.5” represents AMP concentration [mg/mL] in solution) were washed three times for 2 min with sterile-filtered Type II water in an ultrasound bath. Then, to ensure disinfection, samples were incubated twice for 15 min in 70% v/v ethanol (EtOH), rinsed three times with sterile-filtered Type II water, dried with a gentle stream of argon, and stored in sterile 24-well suspension plates (SARSTEDT, Germany) protected from light until further use (either surface characterization or biological assays).

The same protocol was performed to fabricate: MAO-CDI surfaces, representing coatings with conjugated CDI (MAO-CDI∗) but afterwards only incubated in blank borax buffer (without peptide); and MAO/AMP-0.5 surfaces, which were not treated with CDI coupling agent (only incubated in THF), but they were exposed to MSI-78 solution (0.5 mg/mL) for physical AMP adsorption.

#### 2.4.2 Poly(ethylene glycol) (PEG) spacer

The PEG-based conjugation approach is illustrated in Scheme 1B. A heterobifunctional carboxyl-PEG_113_-maleimide spacer molecule (HOOC-PEG-MAL; M_w_ 5000 Da) was purchased from JenKem Technology Co., with purity ≥95%. Three solutions containing individually a (i) HOOC-PEG-MAL spacer, (ii) 1-(3-dimethylaminopropyl)-3-ethylcarbodiimide hydrochloride (EDC; Thermo Scientific) coupling agent, or (iii) 4-(dimethylamino)pyridine (DMAP; Sigma-Aldrich) catalyst were prepared separately in anhydrous dichloromethane as solvent (Thermo Scientific). Then, the solutions (i, ii, and iii) were placed sequentially in contact with the MAO (O_2_ plasma) samples, in order to cover the sample surface completely and attain the final concentration of 1 mg/mL HOOC-PEG-MAL, 10 mg/mL EDC, and 2 mg/mL DMAP. The reaction between MAO (O_2_ plasma) surfaces and the PEG spacer was carried out overnight at RT, 100 rpm and absence of light, in an orbital shaker (IKA KS 3000) (Scheme 1, step B-II). Then, the MAO-PEG∗ samples were rinsed twice with THF, three times with sterile-filtered Type II water, and immediately incubated in peptide solution.

The peptide MSI-78-Cys was used for covalent AMP grafting. Two concentrations of AMP solutions (0.5 and 1 mg/mL) were prepared in a fresh and sterile-filtered phosphate buffer (PB) at pH 6.6. MAO-PEG∗ samples were incubated with peptide solutions for 24 h at RT, 100 rpm, and protected from light in an orbital shaker (IKA KS 3000) (Scheme 1, step B-III). After AMP incubation, MAO-PEG-AMPCys-0.5 and MAO-PEG-AMPCys-1 samples were washed three times for 2 min with sterile-filtered Type II water in an ultrasound bath. Then, the disinfection procedure was carried out as described in Section 2.4.1, to finally store the samples in sterile 24-well suspension plates (SARSTEDT, Germany) in the dark until further use (either surface characterization or biological assays).

The same protocol was performed to fabricate the following samples: MAO-PEG, representing PEGylated MAO surfaces without peptide, being only incubated in blank PB; and MAO-PEG/AMP-0.5, representing PEGylated MAO surfaces treated with the original MSI-78 (0.5 mg/mL) (Scheme 1, step B-IV), *i.e.*, without the additional C-*terminus* cysteine residue to test conditions of physical AMP adsorption.

### 2.5 Surface characterization

#### 2.5.1 Morphology and chemical composition

The microstructure and elemental composition of the surfaces were analyzed by scanning electron microscopy (SEM; Phenom XL G2, Thermo Scientific) and energy dispersive X-ray spectroscopy (EDS; software integrated in Phenom user interface). Surface-sensitive imaging was obtained using a secondary electron (Everhart Thornley) detector, and chemical contrast imaging by a backscattered electron (BSE) detector, within 10-15 kV acceleration voltage. Chemical mappings by EDS were acquired using electron beam at 15 kV.

#### 2.5.2 Wettability

Static water contact angle (WCA) measurements were performed to investigate the wettability behavior of the surfaces, using a goniometer (Data Physics, model OCA15) coupled to a video CCD-camera and SCA20 software. A Type II water 4 μL sessile droplet was dispensed on top of the surfaces, and the resulting drop profiles were calculated through Laplace-Young (contact angles ≥45°) or Tangent methods.

#### 2.5.3 Top surface chemical composition

The top surface of the coatings was evaluated by X-ray photoelectron spectroscopy (XPS; ESCALAB 250Xi, Thermo Fisher Scientific). A monochromatic Al Kα X-ray source (1486.68 eV) was operated at 220 W and 14.6 kV, with a spot size of 400 μm. Charge neutralization was performed, and photoelectrons were collected with a take-off angle of 90°. Pass energies of 100 and 40 eV were set for survey and high-resolution spectra, respectively. In addition, an energy step of 0.1 eV was applied for high-resolution regions. CasaXPS software (version 2.3.19 PR 1.0) was used for data fitting, considering the C 1s peak (284.6 eV) as calibration energy. XPS core levels were quantified by running relative sensitivity factors of the CasaXPS library, and corresponding peak deconvolution was done through Gaussian components and a Shirley-type background.

#### 2.5.4 Indirect quantification of immobilized AMP

The maximum amount of peptide immobilized onto the coatings was estimated indirectly by quantifying the AMP present in the solution before and after incubation with the samples. For that, absorbance of the peptide solutions was measured in a NanoDrop One Micro-UV/Vis spectrophotometer (Thermo Scientific), at 205 nm wavelength using an extinction coefficient of 31 mL mg^−1^cm^−1^. To estimate the maximum surface AMP density, the peptide amount in the incubation solution was subtracted from the initial amount of used AMP. Blank buffer solutions were defined as background control.

#### 2.5.5 Stability tests

Samples were incubated in phosphate buffer under slightly acidic conditions (pH 5.8), for 24 h at 37 °C. After that, surfaces were washed with Type II water, dried at RT, and evaluated by XPS and WCA. Supernatants were also collected to evaluate AMP release using UV/Vis spectrophotometry as described in Section 2.5.4.

Complementary AMP release experiments were carried out in phosphate-buffered saline (PBS, pH 7.4), across a three-day period at 37 °C, with aliquots collected at multiple intervals (5 h, 1 day, 2 days, and 3 days) and measured by UV/Vis spectrophotometry.

### 2.6 Surface antibacterial activity evaluation

#### 2.6.1 Bacterial strains, media, and growth conditions

The antimicrobial activity of the surfaces was tested against methicillin-resistant *S. aureus* (MRSA), obtained from the American Type Culture Collection (ATCC 33591). Bacteria were first grown on Tryptic Soy Agar (TSA; Pronadisa) plate and then overnight in Tryptic Soy Broth (TSB; Merck) at 37 °C, under 150 rpm. PBS (pH 7.4) was used as the suspension medium for the bacterial inoculum.

#### 2.6.2 Bacterial adhesion and viability assays

Before interaction with bacteria, samples in 24-well plates were completely covered with 700 μL human plasma (1% v/v in sterile-filtered PBS) and incubated for 30 min at 37 °C and 150 rpm, for surface protein adsorption as previously reported [65,66]. After incubation with human plasma, samples were gently washed three times with PBS, and readily immersed in bacterial suspension. Human plasma was obtained from surplus buffy coats from healthy blood donors, kindly provided by the *Serviço de Imonuhemoterapia*, *Centro Hospitalar Universitário São João* (CHUSJ), Porto, Portugal. Procedures were approved by the *Centro Hospitalar Universitário São João* Ethics Committee (protocol 90/19) and CECRI-Committee for Ethical and Responsible Conduct of Research of i3S (protocol N4-2025). A written informed consent was obtained from all subjects before sample collection.

To prepare the inoculum, bacteria pre-cultured in TSB were recovered by centrifugation (2700 rpm, 10 min), washed in PBS (pH 7.4), and resuspended in 5 mL of PBS. The inoculum was adjusted to 2x10^5^ colony-forming units (CFU)/mL in PBS, by using optical density (OD) values at 600 nm in PBS. For confirmation of CFU counting, the bacterial inoculum was serially diluted in PBS and inoculated onto TSA plates overnight at 37 °C. Then, samples pre-treated with human plasma were totally submersed in 700 μL bacterial suspension, and incubated for 5 h at 37 °C and 150 rpm.

After bacterial incubation, the viability of surface adherent bacteria was assessed using a LIVE/DEAD® Bacterial Viability Kit (Baclight™ L13152, Invitrogen), as performed before [59,67]. For this purpose, bacterial suspension was removed from the wells, and samples were gently rinsed twice with sterile-filtered aqueous NaCl (0.9% w/v). Afterwards, samples were stained with a combination of two nucleic acid dyes, red-fluorescent propidium iodide (PI) and green-fluorescent Syto9, for 15 min at RT in the dark, according to the manufacturer's instructions. This characterization is based on the bacterial membrane integrity: live cells with an intact membrane will be marked only in green (Syto9); on the other hand, cells with a damaged membrane are considered dead or dying, which allow the entrance of PI and are stained in red. Some bacterial cells may also be observed in yellow, as a result of double-staining from both dyes; since PI must only crossover compromised cell membranes, the yellow-stained bacteria were also assumed as dead [68].

Following the staining procedure, samples were transferred to 24-well black plates with a polymer coverslip bottom (ibid μ-Plates), containing 200 μL of 0.9% w/v NaCl in each well. The testing surface area was placed facing downwards. Then, surface adherent bacteria were observed using a confocal high-content screening system (Opera Phenix Plus) with a 63x/water objective lens, obtaining images of 35 different fields of view (corresponding to a net area of about 1.45 mm^2^ per sample). Both identification and counting of live and dead bacteria were done using Harmony High-Content Imaging and Analysis software (version 5.1). At least three independent assays were carried out, with three replicates each.

#### 2.6.3 Bacterial morphology

To investigate the morphology of surface adherent bacteria, samples were examined by SEM/EDS. For that, the sessile bacteria were fixed with a freshly prepared solution of 1.5% v/v glutaraldehyde (Merck) in 0.14 M aqueous sodium cacodylate (Merck) buffer for 30 min at RT. After rinsing the samples twice with Type II water, adherent bacteria were dehydrated through a graded series of ethanol aqueous concentrations (50, 60, 70, 80, 90, and 99%), keeping them for 10 min in each solution. Afterwards, the samples were covered with hexamethyldisilazane (Sigma) and left to dry overnight. To increase surface conductivity, samples were sputter-coated with gold-palladium before electron imaging.

### 2.7 In vitro osteocompatibility

#### 2.7.1 Cell culture conditions

Human osteoblast-like cells (MG-63 cell line, ATCC CRL-1427™) were cultured in alpha-Minimal Essential Medium (α-MEM) supplemented with 10% v/v fetal bovine serum (FBS), 100 IU/mL penicillin, 100 μg/mL streptomycin, and 0.25 μg/mL amphotericin B (all reagents from Gibco). Cells were maintained at 37 °C in a humidified atmosphere with 5% CO_2_. Upon reaching 70–80% confluence, cells were detached using TrypLE Express (Gibco) for 5 min at 37 °C for subsequent experiments.

#### 2.7.2 Osteoblast adhesion and morphology

MG-63 cells were seeded on samples at a density of 1 × 10^4^ cells/cm^2^ and incubated at 37 °C for 4, 24, and 72 h. At each time point, the medium was removed, samples were gently rinsed with PBS, and adherent cells were fixed with 4% v/v paraformaldehyde (PFA; Sigma-Aldrich) for 15 min. Cell adhesion, morphology, and distribution were analyzed by fluorescence microscopy and SEM. For fluorescence microscopy, fixed cells were permeabilized with 0.1% v/v Triton X-100 (Sigma-Aldrich) in PBS for 15 min, and blocked with 1% w/v bovine serum albumin (BSA; Sigma-Aldrich) in PBS for 30 min to reduce non-specific binding. Adherent cells were then incubated with Phalloidin 488 (1:100; BioLegend) in the dark, overnight at 4 °C and 50 rpm, to stain F-actin (green). After rinsing with PBS, Hoechst 33342 (8 μg/mL; Enzo) was added for 10 min to stain nuclei (blue). Samples were transferred to 24-well black μ-Plates (ibid) and imaged using a confocal high-content screening system (Opera Phenix Plus) with a 20x/water objective lens. Images were acquired from 17 distinct regions, covering an area of approximately 6.98 mm^2^ per sample.

For SEM analysis (Phenom XL G2, Thermo Scientific), fixed cells were dehydrated in increasing concentrations of ethanol aqueous solutions for 10 min each and subjected to critical point drying (CPD 7501, Polaron Range). To improve surface conductivity, specimens were sputter-coated with gold-palladium before electron imaging.

#### 2.7.3 Osteoblast viability and metabolic activity

MG-63 cells were seeded onto the samples at a density of 3 × 10^4^ cells/cm^2^ and incubated at 37 °C for up to 15 days.

Cell viability was assessed after 24 h using Live/Dead staining. Samples were carefully rinsed with PBS, and adherent cells were incubated in the dark with two fluorophores: calcein acetoxymethyl (Calcein-AM; 1:1000; BioLegend) and PI (0.55 μM; Baclight™ L7012, Invitrogen) for 15 min at 37 °C. Calcein-AM, converted into green-fluorescent calcein by live cells, indicates viable cells, while PI, which enters only damaged membranes, marks dead cells in red. Stained cells were imaged using the Opera Phenix Plus equipment with a 10x/water objective lens. Images were obtained from 25 different zones, corresponding to an area of about 41.1 mm^2^ per sample.

Cell metabolic activity was evaluated using the resazurin assay on days 1, 3, 8, 11, and 15. At each time point, samples were transferred to new wells and incubated with fresh medium containing 10% v/v resazurin solution (0.1 mg/mL; Sigma-Aldrich) for 3 h at 37 °C. Fluorescence of the supernatant was measured using a microplate reader (Synergy HT, Biotek) at 530/590 nm (excitation/emission). Two independent experiments with five replicates per condition were carried out.

### 2.8 In vitro osteogenic behavior

#### 2.8.1 Cell culture conditions

Human mesenchymal stem cells (hMSC) (Lonza, Catalog #: PT-2501) were used to evaluate cytocompatibility and osteogenic response. Cells were maintained in basal culture medium as outlined in Section 2.7.1. Osteogenic differentiation was induced by supplementing the basal medium with 10 mM β-glycerophosphate, 50 μg/mL ascorbic acid, and 10 nM dexamethasone (Sigma-Aldrich).

hMSCs were seeded onto the samples at a density of 1 × 10^4^ cells/cm^2^ in both basal and osteogenic media. Cultures were incubated at 37 °C for up to 21 days, with medium replenished every three days. In parallel, cells seeded onto tissue culture-treated wells served as controls.

#### 2.8.2 hMSC metabolic activity

Cell metabolic activity was evaluated over a 21-day culture period using the resazurin assay, as described in Section 2.7.3.

#### 2.8.3 hMSC morphology

The morphology of adherent cells under basal conditions was evaluated by fluorescence microscopy, whereas morphology under osteogenic conditions was assessed by SEM, using the protocols detailed in Section 2.7.2. For fluorescence imaging, stained cells were examined using a SP5 confocal microscope (Leica Microsystems, Germany) equipped with 10x and 63x water-immersion objective lenses.

#### 2.8.4 Alkaline phosphatase (ALP) activity

The osteogenic response of hMSCs cultured on the samples under osteogenic conditions was further evaluated by measuring ALP activity. ALP activity was quantified based on substrate hydrolysis in an alkaline environment (pH 10.5). Briefly, *p*-nitrophenyl phosphate (Sigma-Aldrich) was dephosphorylated by ALP, yielding *p*-nitrophenol, the concentration of which was determined using a microplate reader (Synergy HT, Biotek) at 405 nm. ALP activity was normalized to total protein content, determined using the DC™ Protein Assay (Bio-Rad) according to the manufacturer's protocol. Results were expressed as a percentage relative to the control group (*i.e.*, cells cultured on tissue culture-treated wells), which was defined as 100%.

### 2.9 Statistical analysis

Statistical analyses were performed using GraphPad Prism software (version 8.0.2). When data met the normality assumptions, comparisons among multiple experimental groups were conducted using one-way ANOVA followed by Tukey's post hoc test. For analyses that included a control group, one-way ANOVA followed by Dunnett's post hoc test was applied. For data that did not follow normal distribution, Kruskal−Wallis non-parametric tests with corrected Dunn's multiple comparison test were done. Data were expressed as mean ± standard deviation (SD), and p values of <0.05 were considered statistically significant.

## 3 Results

### 3.1 Molecule immobilization strategies onto MAO surfaces

MAO surfaces were first activated with hydroxyl (-OH) species by O_2_ plasma [64], to allow the reaction either with CDI coupling agent or the heterobifunctional HOOC-PEG-MAL spacer.

The CDI-based conjugation approach was conducted in two sequential reactions (please, see Scheme 1A), as previously described [55,56]. First, the surface -OH groups of the MAO (O_2_ plasma) coatings were reacted with the electrophile carbonyl group of the CDI molecule (Scheme 1A, step II). Then, MSI-78 was grafted onto the surface through the reaction between one of the free amine groups (-NH_2_) from the peptide and the carbonyl of the intermediate MAO-CDI∗ (Scheme 1A, step III), resulting in MAO-CDI-AMP-0.5 samples.

The PEG-based conjugation approach was also performed in two reactions (please, see Scheme 1B). Through the use of EDC as coupling agent and DMAP as catalyst, the surface -OH groups of the MAO (O_2_ plasma) coating reacted with the carboxyl from the HOOC-PEG-MAL spacer (Scheme 1B, step II). Then, MSI-78-Cys was grafted onto the surface through a Michael Addition reaction [59]: the exposed maleimide (MAL) group from the PEG reacted with the thiol (-SH) group from the cysteine, present in the peptide MSI-78-Cys (Scheme 1B, step III). The resulting functionalized surfaces were MAO-PEG-AMPCys-0.5 and MAO-PEG-AMPCys-1.

Apart from these covalent AMP grafting approaches, conditions of physical AMP adsorption were also analyzed. These include MSI-78 adsorbed onto MAO surfaces (MAO/AMP-0.5) and PEGylated MAO surfaces (MAO-PEG/AMP-0.5).

### 3.2 Characterization of the surfaces

#### 3.2.1 Morphology and chemical composition

**Fig. 1:**
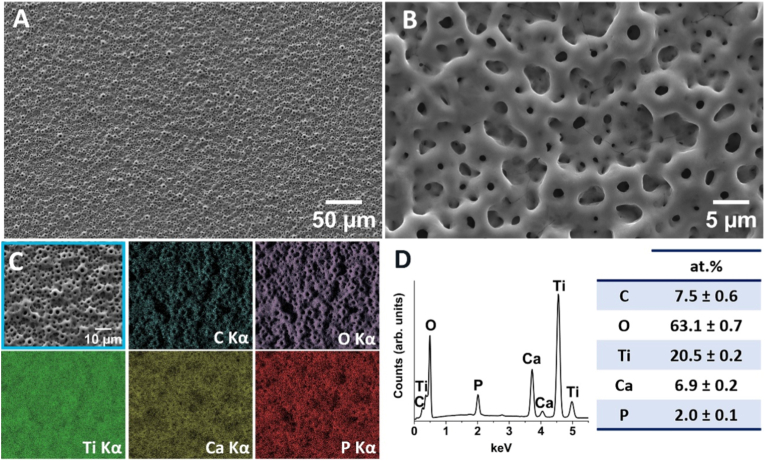
Morphology (SEM images) and chemical composition (EDS analysis) of the MAO-control coatings. Surface morphology at (A) lower and (B) higher magnification. Elemental EDS mapping taken from region (C), for carbon (C Kα), oxygen (O Kα), titanium (Ti Kα), calcium (Ca Kα), and phosphorus (P Kα). (D) Representative EDS spectrum with corresponding atomic concentrations (*n* = 3).

Morphology (SEM) and chemical composition (EDS) of MAO-control samples are shown in Fig. 1. The surface presented volcano-like structures, with pores of distinct sizes, ranging from the nanometer scale to a few micrometers, due to the energetic discharges created over the surface during MAO process [64]. These TiO_2_ coatings were doped with carbon (C), calcium (Ca), and phosphorus (P) with uniform distribution on the surface. No significant alterations in surface morphology and chemical composition (EDS) were detected following the AMP immobilization methods (data not shown).

#### 3.2.2 Wettability

**Fig. 2:**
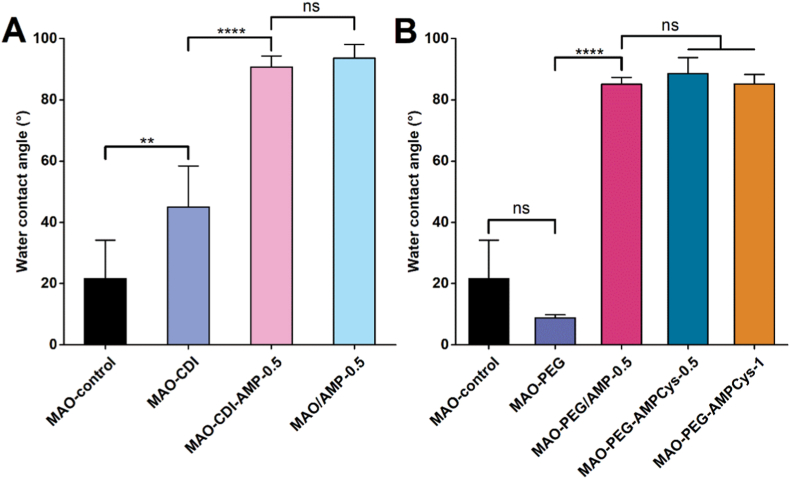
WCA measurements of the surfaces prepared using (A) CDI coupling and (B) PEG spacer. Data are presented as mean ± SD (∗∗p < 0.01, ∗∗∗∗p < 0.0001, ns: not significant) (*n* = 3).

The wettability of the coatings before and after surface modification is presented in Fig. 2. For the CDI coupling strategy (Fig. 2A), a significant increase in WCA was noticed for MAO-CDI samples (45 ± 13°) compared to MAO-control coatings (22 ± 13°) (p < 0.01), suggesting successful surface functionalization with CDI [55,56]. Hydrophobicity is further increased following peptide immobilization, with WCA values reaching approximately 90° (p < 0.0001 compared to MAO-CDI). However, no significant difference in WCA was observed between samples with peptides covalently grafted (MAO-CDI-AMP-0.5) and physically adsorbed (MAO/AMP-0.5).

Regarding the PEG-based conjugation strategy (Fig. 2B), the introduction of the PEG chain (MAO-PEG) did not result in statistically significant changes in WCA relative to the MAO-control; however, these surfaces presented a more stable hydrophilic behavior (9 ± 1°). After AMP immobilization onto MAO-PEG surfaces, a significant increase in WCA was observed for all the samples, independently of the immobilization method or peptide concentration (∼85°).

#### 3.2.3 Top surface chemical composition

**Fig. 3:**
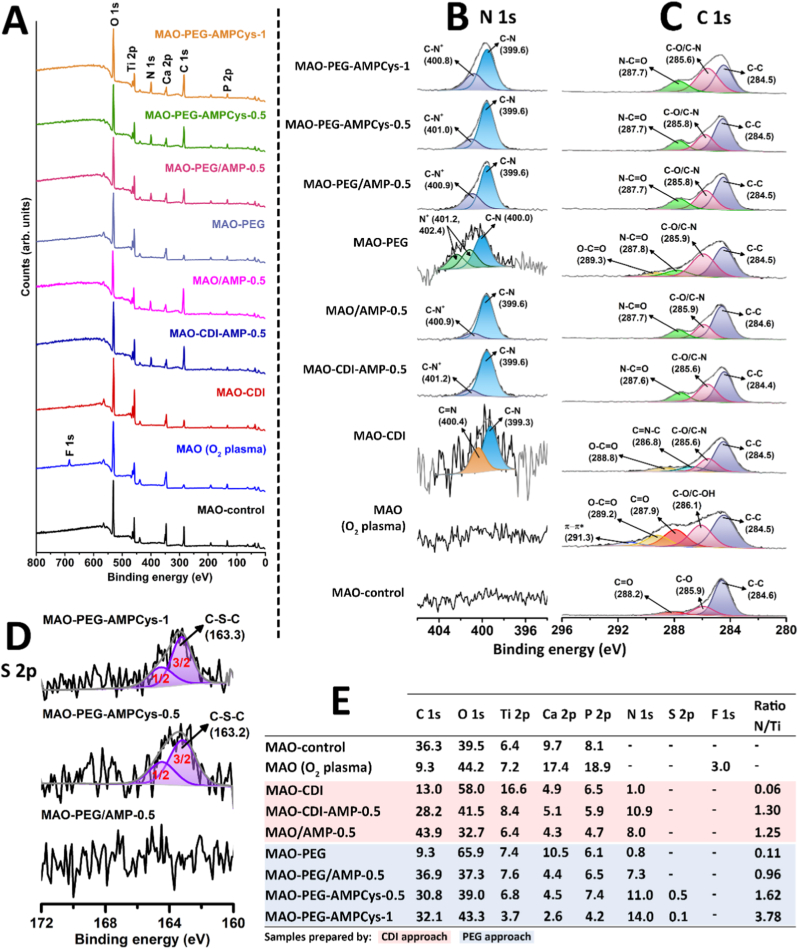
XPS analysis of surfaces prepared using different AMP immobilization strategies. (A) Wide XPS spectra (survey). High resolution XPS spectra of the (B) N 1s, (C) C 1s, and (D) S 2p regions, together with possible contributions assigned to the fitted peaks (binding energies in eV are given in parentheses). (E) Surface atomic composition (%) calculated from the high resolution XPS spectra.

XPS analyses of the surfaces are presented in Fig. 3. Survey scans (Fig. 3A) reveal the presence of titanium (Ti 2p), oxygen (O 1s), calcium (Ca 2p), and phosphorus (P 2p) across all samples. These core peaks at high resolution indicated the formation of TiO_2_ coatings containing calcium carbonate and calcium phosphate compounds (data not shown), as reported for MAO coatings elsewhere [48,64]. Fluorine was detected in MAO (O_2_ plasma) samples, possibly due to contamination from the cleaning chamber; nevertheless, the F 1s signal disappears after molecule immobilization procedures. The treatment with peptides (whether via covalent grafting or physical adsorption) resulted in an obvious introduction of N 1s signals, implying successful AMP immobilization.

Looking at the high resolution N 1s spectra (Fig. 3B), all peptide-treated surfaces showed C–N species at 399.6 eV that can be assigned to typical peptide bonds, as well as C–N^+^ peaks around 401 eV that may be attributed to protonated nitrogen species from amino acids (lysine, K) [[69], [70], [71]]. Regarding the coupling molecules, MAO-CDI surfaces exhibited C–N contributions at 399.3 eV, and C=N groups at 400.4 eV [72], suggesting the presence of some remaining imidazole groups from CDI, which are sensitive to further hydrolysis events in the buffer [55,56]. The N 1s envelope of MAO-PEG samples was fitted into three peaks, indicating the immobilization of the heterobifunctional spacer on the surface: the binding energy at 400.0 eV from C–N bonds [71]; 401.2 and 402.4 eV that both may suggest N^+^ species [70,71,73].

In the C 1s region (Fig. 3C), both MAO-CDI and MAO-PEG samples presented O–C=O assignments (288.8 – 289.3 eV) [64,70,74] which may be related to the linkage of the coupling/spacer molecule to the surface, as well as some remaining COO^−^ groups from the activation by O_2_ plasma. Another indication of coupling/spacer immobilization relies on the formation of C=N–C (286.8 eV) [75,76] and N–C=O (287.8 eV) [64,77,78] bonds for MAO-CDI and MAO-PEG samples, respectively. This same contribution (N–C=O) was detected after peptide immobilization using the different strategies, corroborating the presence of AMP on the surface, together with C–O/C–N (285.6 – 285.9 eV) and C–C (284.4 – 284.6 eV) components [64].

The sulfur molecular environments (S 2p) at high resolution are presented in Fig. 3D. The success of covalent peptide grafting was further suggested by the appearance of C–S–C bonds (163.2 – 163.3 eV) [67,74,79] for both MAO-PEG-AMPCys-0.5 and MAO-PEG-AMPCys-1 surfaces, due to the reaction between -SH groups from the peptide and the exposed maleimide from the PEG spacer. MAO-PEG/AMP-0.5 samples did not present contributions due to the absence of cysteine in the original peptide MSI-78.

The elemental contents calculated from high resolution XPS spectra are shown in Fig. 3E. Following O_2_ plasma treatment, MAO coatings exhibited a reduction in surface carbon, which may have led to increased exposure of bioactive Ca and P elements, as previously reported [64]. For peptide-treated samples, the remarkable increase in C 1s and N 1s contents may be accompanied by the decrease of Ti 2p and Ca 2p/P 2p core levels, as a result of the formation of peptide layer covering the surface. As the matrix material of the MAO coating is TiO_2_, we have assessed the N/Ti ratios. MAO-CDI surfaces presented an N/Ti ratio of 0.06, which was highly increased to 1.30 in MAO-CDI-AMP-0.5 samples. However, this covalent peptide grafting condition through CDI coupling was not different from peptides physically adsorbed, showing a ratio of 1.25 for MAO/AMP-0.5.

MAO-PEG surfaces presented an N/Ti ratio of 0.11, rising to 0.96 after adsorbing peptides (MAO-PEG/AMP-0.5). In direct comparison with the covalent grafting strategy using the same peptide concentration in solution (0.5 mg/mL), the MAO-PEG-AMPCys-0.5 showed a higher proportion, reaching 1.62. This value was then further expanded to 3.78 for MAO-PEG-AMPCys-1 samples.

#### 3.2.4 AMP immobilization quantification

Surface peptide densities, indirectly estimated by UV/Vis spectrophotometry, are presented in Table 1. For the CDI coupling strategy, similar AMP densities were found after covalent grafting (MAO-CDI-AMP-0.5) and physical adsorption (MAO/AMP-0.5). The AMP covalent grafting was further increased through the PEG molecules, reaching the highest value for MAO-PEG-AMPCys-1. In contrast, when AMP was only adsorbed, MAO-PEG/AMP-0.5 samples showed the lowest surface AMP density.

**Table 1:** Surface peptide densities, indirectly estimated by UV/Vis spectrophotometry, for the samples prepared by the different AMP immobilization strategies (*n* = 2).

Samples	Estimated surface AMP density (μg/mm^2^)
MAO-CDI-AMP-0.5	0.5 ± 0.2
MAO/AMP-0.5	0.6 ± 0.2
MAO-PEG-AMPCys-0.5	1.0 ± 0.3
MAO-PEG-AMPCys-1	2.0 ± 0.5
MAO-PEG/AMP-0.5	0.2 ± 0.1

#### 3.2.5 AMP-treated coating stability

The stability of AMP immobilization was assessed after 24 h incubation in a slightly acidic environment (PB, pH 5.8). No detectable release of peptides into the supernatants was observed by UV/Vis spectrophotometry (sensitivity 1 μg/mL), regardless of the AMP immobilization strategies.

**Fig. 4:**
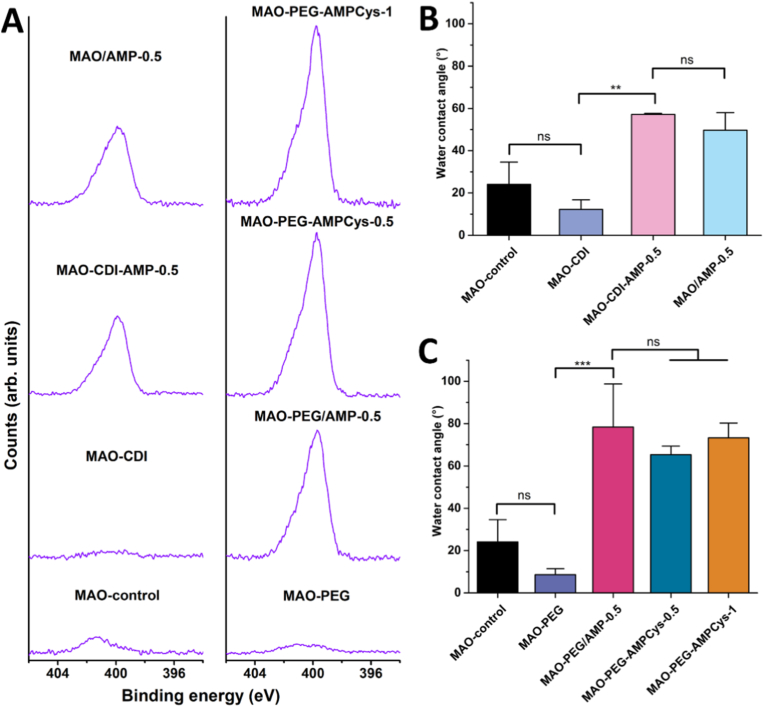
Surface analysis after immersion under slightly acidic conditions (PB, pH 5.8, for 24 h at 37 °C). (A) High resolution N 1s XPS spectra and (B, C) WCA measurements of the surfaces prepared by the different AMP immobilization strategies. Data are presented as mean ± SD (∗∗p < 0.01, ∗∗∗p < 0.001, ns: not significant) (*n* = 3).

Post-incubation surface analysis by XPS and WCA is presented in Fig. 4. The high intensity of N 1s signals confirms the retention of peptides after the acidic incubation (peak deconvolution was similar to Fig. 3B, and it was included as Fig. S1 in Supplementary Material). Furthermore, all AMP-treated coatings maintained their hydrophobic character, independent of the immobilization method employed.

Additional AMP release profiling of the MAO-PEG/AMP-0.5 surfaces was performed during incubation in PBS (pH 7.4) at 37 °C over three days. Notably, UV/Vis spectrophotometric measurements indicated an absence of detectable AMP release at the monitored intervals (5, 24, 48, and 72 h).

### 3.3 Surface antibacterial activity

**Fig. 5:**
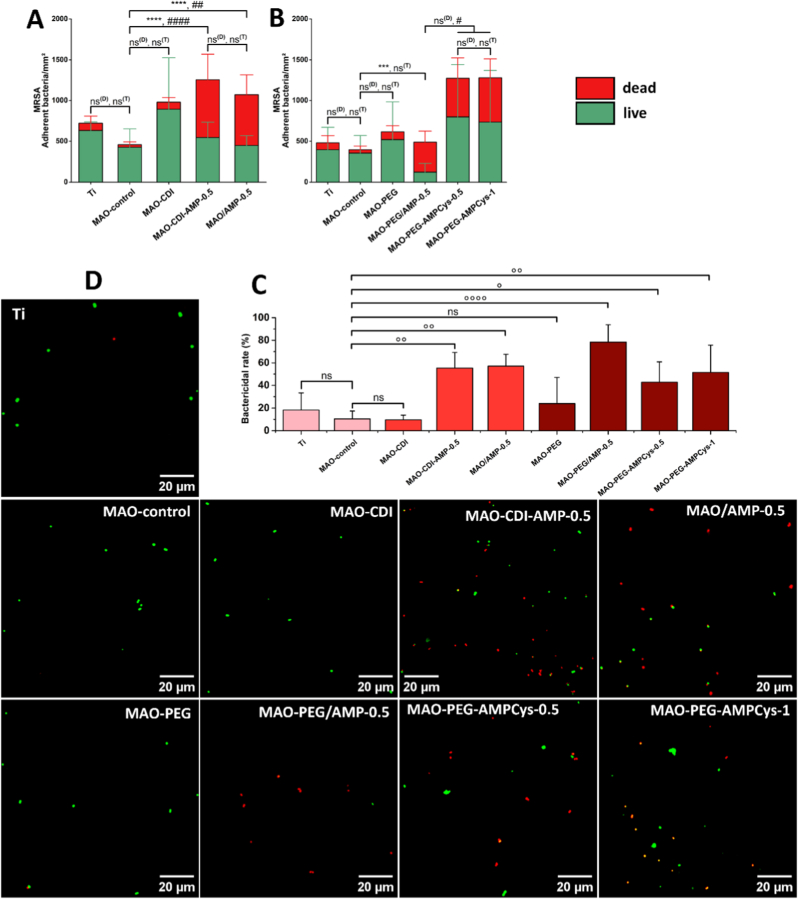
Viability of adhered MRSA after incubation for 5 h on surfaces prepared by (A) CDI coupling and (B) PEG spacer, together with (C) bactericidal rate (%) and (D) representative fluorescence staining images. Bare titanium substrates (Ti) and MAO-control samples were also tested. Before bacterial incubation, all surfaces were pre-treated with human plasma proteins (1% v/v) for 30 min. Data are presented as mean ± SD. Statistical analysis refers to: the number of dead bacteria (∗∗∗p < 0.001, ∗∗∗∗p < 0.0001, ns^(D)^: not significant); the total number of adherent bacteria (live + dead cells; #p < 0.05, ##p < 0.01, ####p < 0.0001, ns^(T)^: not significant); the bactericidal rate (%) for adherent bacteria (° p < 0.05, °° p < 0.01, °°°° p < 0.0001, ns: not significant) (*n* = 9 for (A) and *n* = 15 for (B)).

The antimicrobial performance of AMP-functionalized coatings against MRSA in the presence of human plasma proteins is shown in Fig. 5, based on live/dead staining to assess both bacterial adhesion and viability. Quantitative data on adherent bacteria are presented in Fig. 5A and B for coatings prepared using CDI coupling and PEG spacer, respectively. Bactericidal rate (%) for adherent bacteria and representative fluorescence microscopy images are shown in Fig. 5C and D, respectively.

Bare Ti substrates and MAO-control coatings exhibited no significant differences in terms of the number of dead cells (ns^(D)^) or the total bacterial adhesion (ns^(T)^). Likewise, the introduction of coupling/spacer molecules (MAO-CDI and MAO-PEG samples) did not affect MRSA adhesion or viability when compared to MAO-control surfaces.

In contrast, surfaces functionalized with peptide grafting via CDI coupling (MAO-CDI-AMP-0.5) and peptide adsorption (MAO/AMP-0.5) presented similar antimicrobial performance (about 55% of dead cells), demonstrating a significantly higher bactericidal rate relative to the MAO-control (p < 0.01). However, these coatings also exhibited increased bacterial adhesion, with significantly higher numbers than the MAO-control (p < 0.0001 for MAO-CDI-AMP-0.5, and p < 0.01 for MAO/AMP-0.5).

Among the tested surfaces, the MAO-PEG/AMP-0.5 coating, in which AMP were physically adsorbed onto a PEGylated MAO surface, exhibited the most favorable antimicrobial profile (78 ± 15% bactericidal rate). This surface maintained bacterial adhesion at levels comparable to the MAO-control, while achieving a significantly higher proportion of dead cells (p < 0.001). In contrast, covalent AMP grafting using a PEG spacer (MAO-PEG-AMPCys-0.5 and MAO-PEG-AMPCys-1 samples) did not improve bactericidal activity and was associated with increased MRSA adhesion compared to MAO-PEG/AMP-0.5 (p < 0.05). Moreover, increasing the surface AMP density using PEG-mediated covalent grafting did not yield any improvement in antimicrobial performance, reaching average bactericidal rate of 51 ± 24%.

**Fig. 6:**
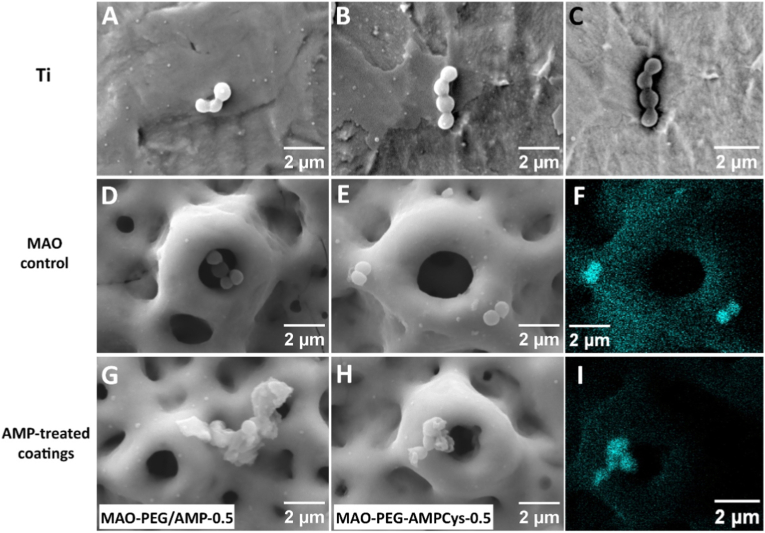
SEM images of MRSA adherent to (A-C) Ti substrates, (D-F) MAO-control coatings, and (G-I) AMP-functionalized coatings. (G) shows surfaces with physically adsorbed AMP (MAO-PEG/AMP-0.5 samples), while (H) and (I) correspond to surfaces with covalently grafted AMP (MAO-PEG-AMPCys-0.5 samples). Chemical information is provided for panel B, through (C) atomic contrast using a BSE detector, and for panels E and H, through (F, I) carbon content (C Kα) using EDS mapping.

Surfaces were examined by SEM after MRSA adhesion (Fig. 6) to gain further insight into the mechanism of action of the peptide-functionalized coatings. Carbon accumulation from bacterial content was visually reinforced by atomic contrast in the BSE image (Fig. 6C) and confirmed by EDS mapping (Fig. 6F–I).

Titanium substrate (Fig. 6A–C) and MAO-control coating (Fig. 6D–F) have shown adherent MRSA with the characteristic spherical morphology and no apparent structural disruption. In particular, the Ca/P-doped porous coatings showed bacterial colonization at different sites, including around the pores, as well as almost penetrating such cavities. The introduction of spacer molecules (MAO-PEG samples) did not noticeably alter bacterial morphology (data not shown).

In contrast, surfaces functionalized with AMP, either by physical adsorption (MAO-PEG/AMP-0.5 samples, Fig. 6G) or covalent grafting (MAO-PEG-AMPCys-0.5 samples, Fig. 6H–I), revealed clear morphological damage to MRSA. These included deformation of the bacterial shape and membrane disruption [80], which may lead to the leakage and agglomeration of cell contents. These observations suggest a potent and irreversible bactericidal action of the AMP, likely acting across multiple levels of the coating's surface topography. Surfaces functionalized by the CDI coupling strategy were not investigated by SEM after bacterial adhesion.

### 3.4 In vitro osteocompatibility

**Fig. 7:**
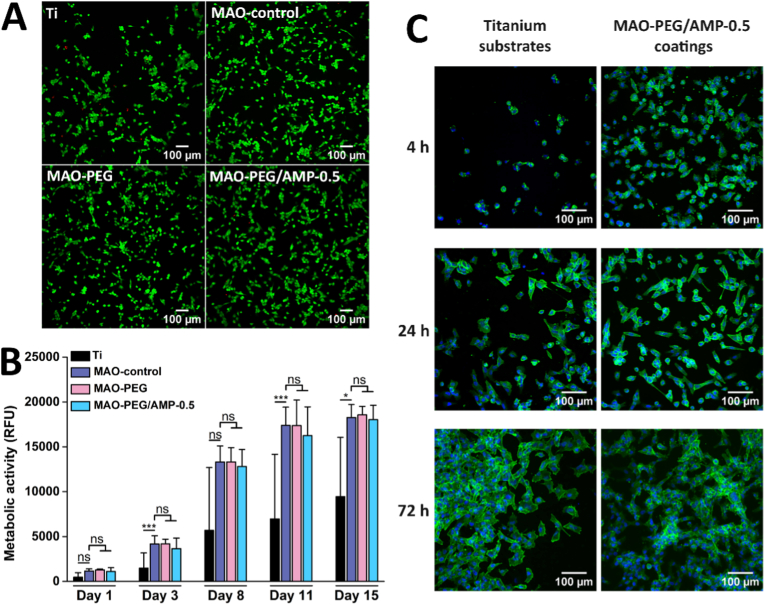
*In vitro* cytocompatibility assays with MG-63 osteoblastic cells: (A) Representative fluorescence images from Live/Dead assay after 24 h of culture on Ti, MAO-control, MAO-PEG, and MAO-PEG/AMP-0.5 surfaces. Viable cells are stained green, while non-viable cells are stained red, indicating compromised membrane integrity. (B) Metabolic activity of cells cultured on the same surfaces over 1, 3, 8, 11, and 15 days, measured as relative fluorescence units (RFU) using the resazurin assay. Data are presented as mean ± SD (∗p < 0.05, ∗∗∗p < 0.001, ns: not significant) (*n* = 9). (C) Fluorescence images of cells cultured for 4, 24, and 72 h on Ti and MAO-PEG/AMP-0.5 surfaces. F-actin filaments are stained green, and cell nuclei are stained blue, illustrating cytoskeletal organization and cell spreading over time. (For interpretation of the references to colour in this figure legend, the reader is referred to the Web version of this article.)

The osteocompatibility of the coatings was evaluated using MG-63 osteoblast-like cells cultured on Ti, MAO-control, MAO-PEG, and MAO-PEG/AMP-0.5 surfaces, the latter selected based on its superior antimicrobial performance (Fig. 7).

Cell viability was first evaluated by live/dead staining after 24 h of incubation (Fig. 7A). Osteoblasts adhered to all surfaces and exhibited high viability, indicating that PEGylated MAO surfaces (MAO-PEG) and PEGylated MAO surfaces with adsorbed AMP (MAO-PEG/AMP-0.5) did not compromise cell viability.

Metabolic activity, assessed using the resazurin assay over 15 days (Fig. 7B), showed an upward trend, particularly for the porous coatings (MAO-control, MAO-PEG, and MAO-PEG/AMP-0.5), which plateaued around day 11, as evidenced by stabilized RFU values through day 15. Metabolic activity on porous coatings was more consistent and stable than on bare Ti substrates. Osteoblasts on MAO-control surfaces exhibited significantly higher viability/proliferation than those on Ti at days 3, 11, and 15 (∗∗∗p < 0.001, ∗∗∗p < 0.001, and ∗ p < 0.05, respectively). No significant differences were detected among MAO-control, MAO-PEG, and MAO-PEG/AMP-0.5 coatings, suggesting that the surface functionalization was not detrimental for osteoblast and, in fact, enhanced osteoblast proliferation compared to bare Ti.

Cell morphology and spreading were further examined through immunofluorescence staining of the F-actin cytoskeleton and nuclei at 4, 24, and 72 h of culture (Fig. 7C). Representative images are shown for Ti and MAO-PEG/AMP-0.5 surfaces, as the MAO-control and MAO-PEG samples showed comparable morphologies to MAO-PEG/AMP-0.5. At 4 h, osteoblasts on all surfaces displayed a rounded morphology with limited cytoplasmic extension, particularly on Ti. A marked difference in the number of cells was also observed between the surfaces. Over time, a significant increase in cell density and spreading was evident. By 24 h, cells had colonized a larger surface area, adopting an elongated morphology and developing cytoplasmic projections, indicating active surface adaptation. By 72 h, cell morphology became consistent across all surfaces, showing increased intercellular connections and a well-organized network of cytoplasmic extensions.

**Fig. 8:**
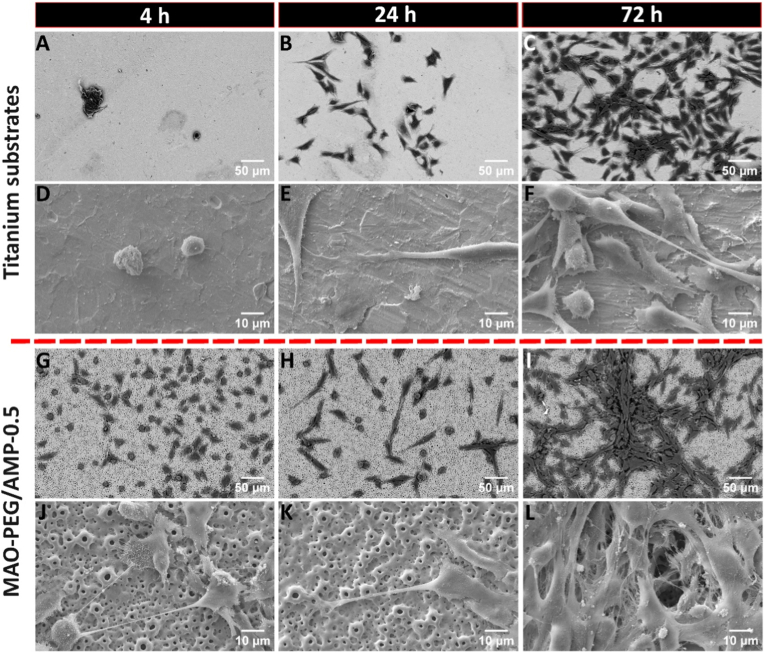
SEM images of MG-63 osteoblastic cells adhered to Ti substrates and MAO-PEG/AMP-0.5 coatings, after 4, 24, and 72 h of culture. (A-C) and (G-I): low magnification images acquired in BSE mode, highlighting overall cell distribution and surface coverage. (D-F) and (J-L): high magnification images acquired in secondary electron mode, detailing cell morphology, adhesion structures, and interactions with the underlying surface topography.

SEM detailed osteoblast adhesion at 4, 24, and 72 h of incubation (Fig. 8) for Ti and MAO-PEG/AMP-0.5 surfaces. MAO-control and MAO-PEG surfaces showed consistent adhesion patterns with those of MAO-PEG/AMP-0.5 (data not shown). After 4 h, osteoblasts on the Ti substrate displayed an initial adhesion characterized by a rounded morphology and limited cytoplasmic extensions (Fig. 8A and D). In contrast, cells on MAO-PEG/AMP-0.5 coatings showed more advanced adhesion, with pronounced filopodia and lamellipodia formation (Fig. 8G and J). The porous structure of these coatings provided an anchoring platform, facilitating cytoplasmic projections that extended across and into the pores. Cell bodies also established intercellular contact via long membrane extensions. By 24 h, osteoblasts on Ti substrate had developed lamellipodia and showed improved spreading (Fig. 8B and E). On MAO-PEG/AMP-0.5 surfaces, cells appeared more elongated, using the volcano-like structures as adhesion points (Fig. 8H and K). By 72 h, both Ti (Fig. 8C and F) and MAO-PEG/AMP-0.5 (Fig. 8I and L) surfaces exhibited significant cell coverage. Numerous cytoplasmic and well-spread morphologies indicated advanced adhesion, cytoskeletal organization, and proliferation across the surface.

### 3.5 In vitro osteogenic behavior

**Fig. 9:**
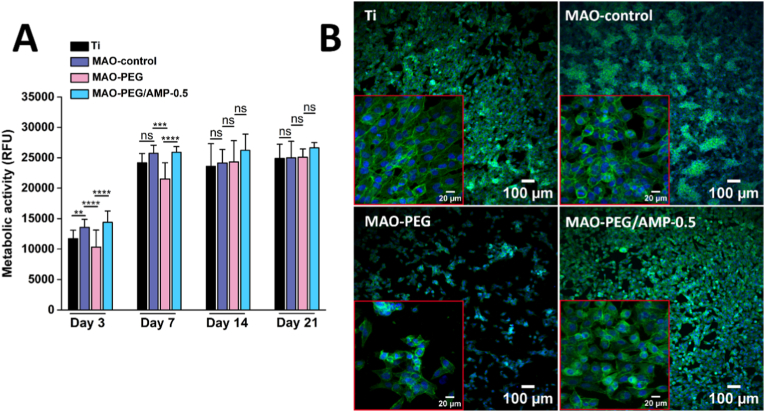
*In vitro* cytocompatibility of hMSCs under basal conditions. (A) Metabolic activity of hMSCs cultured on Ti, MAO-control, MAO-PEG, and MAO-PEG/AMP-0.5 surfaces for 3, 7, 14, and 21 days, quantified as relative fluorescence units (RFU) using the resazurin assay. Data are presented as mean ± SD (∗∗p < 0.01, ∗∗∗p < 0.001, ∗∗∗∗p < 0.0001, ns: not significant) (*n* = 5). (B) Representative fluorescence images of hMSCs cultured for 72 h on the same surfaces. F-actin filaments are stained green, and cell nuclei are stained blue, illustrating cytoskeletal organization and cell spreading. (For interpretation of the references to colour in this figure legend, the reader is referred to the Web version of this article.)

The ability of hMSCs to adhere and proliferate on the different treated surfaces was initially evaluated under basal conditions over a 21-day culture period. As depicted in Fig. 9A, the resazurin assay showed a marked increase in hMSC metabolic activity from day 3 to day 7 across all surfaces, followed by a plateau between days 14 and 21. At the early stage of culture (3 days), hMSCs cultured on MAO-control surfaces exhibited higher metabolic activity than those on bare Ti substrates. For 7 days, MAO-PEG surfaces exhibited statistically lower RFU values compared with MAO-control and MAO-PEG/AMP-0.5 surfaces, suggesting that the introduction of the PEG polymer may affect early cell behavior. In contrast, functionalization with adsorbed AMP supported favorable hMSC metabolic activity. From day 14 onward, no significant differences were observed among the different surfaces, indicating adequate cytocompatibility in terms of both cell adhesion and proliferation.

Additionally, cellular morphology was assessed at day 3 of culture (Fig. 9B), corroborating that initial hMSC adhesion was compromised on MAO-PEG surfaces. Conversely, increased cell density and enhanced spreading were observed on Ti, MAO-control, and MAO-PEG/AMP-0.5 surfaces, characterized by consistent intercellular connections and well-defined cytoplasmic projections.

**Fig. 10:**
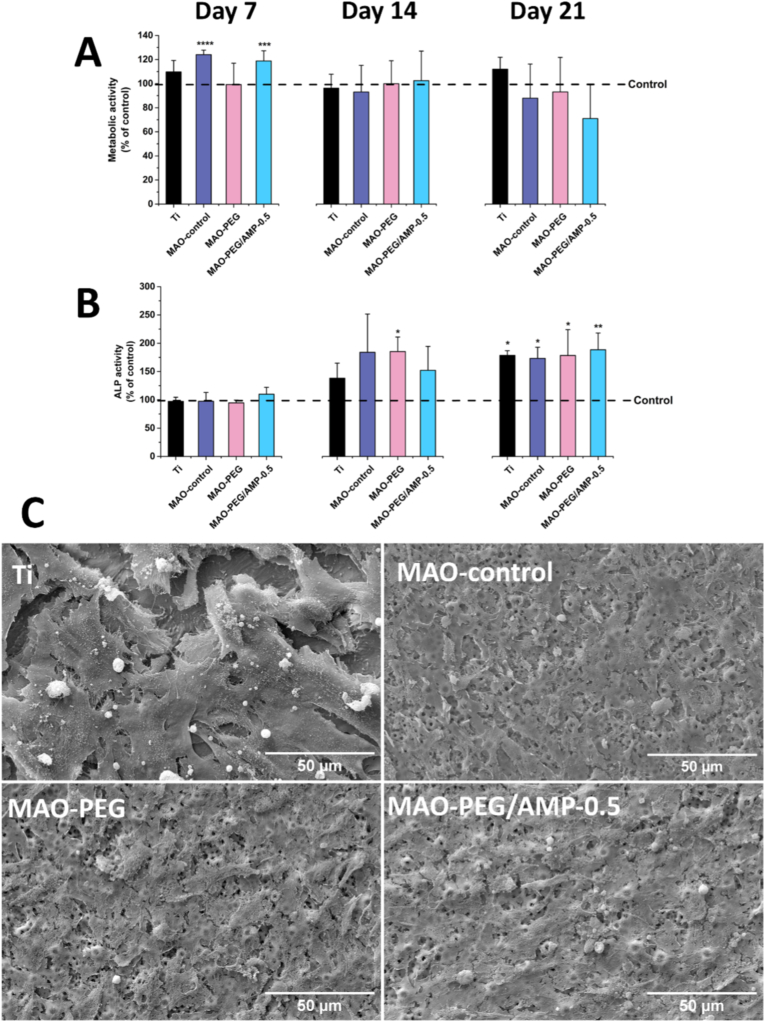
*In vitro* assays with hMSCs under osteogenic conditions. (A) Metabolic activity of hMSCs cultured on Ti, MAO-control, MAO-PEG, and MAO-PEG/AMP-0.5 surfaces for 7, 14, and 21 days, evaluated by resazurin assay and normalized to the control cells (hMSCs cultured on tissue culture-treated wells). (B) ALP activity of hMSCs cultured on the same surfaces for 7, 14, and 21 days. Values were normalized to total protein content and expressed as a percentage relative to the control group. Data are presented as mean ± SD (∗p < 0.05, ∗∗p < 0.01, ∗∗∗p < 0.001, ∗∗∗∗p < 0.0001) (*n = 5* for (A) and *n* = 3 for (B)). (C) Representative SEM images of hMSCs adhered to the surfaces after 21 days of osteogenic culture, illustrating cell spreading and surface coverage.

The metabolic activity of hMSCs cultured on the different material surfaces was also evaluated under osteogenic conditions for up to 21 days. RFU values were normalized to control cells (*i.e.*, cells cultured on tissue culture-treated wells, set at 100%) and are presented in Fig. 10A. At day 7, only cells on the MAO-control and MAO-PEG/AMP-0.5 surfaces exhibited a significant increase in metabolic activity compared to the control group (p < 0.0001 and p < 0.001, respectively). By day 14, no statistically significant differences were observed among the surfaces, with all groups exhibiting metabolic activity similar to that of the control. At day 21, metabolic activity on all material surfaces remained comparable to control levels, with only a slight, non-significant decrease observed on the MAO-PEG/AMP-0.5 surface. Collectively, these findings indicate that none of the surface treatments substantially affected the metabolic activity of hMSCs under osteogenic conditions over the 21-day culture period.

To further assess the osteogenic potential of the treated surfaces, ALP activity of hMSCs was evaluated after 7, 14, and 21 days of culture (Fig. 10B), with all values normalized to the control condition. At day 7, ALP activity was comparable across all experimental groups. By day 14, an overall increase in ALP activity was observed for all materials, with the MAO-PEG surface exhibiting a statistically significant increase relative to control (p < 0.05). By day 21, ALP activity was significantly higher for all surfaces compared to the control, with cells cultured on MAO-PEG/AMP-0.5 exhibiting the highest mean ALP activity (p < 0.01).

hMSC morphology on the materials after 21 days of osteogenic induction was also examined by SEM (Fig. 10C). All surfaces, Ti, MAO-control, MAO-PEG, and MAO-PEG/AMP-0.5 surfaces, supported the formation of a confluent cell layer, with well-spread cytoplasmic extensions closely conforming to the underlying surface topography.

## 4 Discussion

This study explored different strategies for MSI-78 immobilization, an antimicrobial peptide (AMP), onto Ca/P-doped porous TiO_2_ surfaces fabricated by micro-arc oxidation (MAO surfaces). These MAO surfaces have garnered considerable attention in the biomedical field (and other applications), as extensively reviewed in the literature [36,37,[42], [43], [44],^53^,^81^], and are already commercialized by several companies involved in Ti-based implant technologies [52].

However, AMP immobilization onto MAO coatings remains unreported. Unlike passive or sol-gel/sputter-derived TiO_2_ films, MAO coatings are characterized by heterogeneous porosity, Ca/P doping, and mixed TiO_2_ phases (anatase and rutile) [64] – all of which may contribute to complex AMP immobilization behavior.

Therefore, physical AMP adsorption and covalent AMP grafting were evaluated. To achieve a successful covalent immobilization, the first challenge was the activation of the MAO surfaces. To generate surface -OH species, an O_2_ plasma-based protocol was selected due to its simplicity and non-destructive character, as previously investigated by us [64].

The next task was the choice of molecules for conjugation. CDI and PEG-mediated anchoring approaches have been efficiently applied to different target substrates, as self-assembled monolayers (SAMs) [[55], [56], [57], [58]], chitosan [59,60], and even titanium [20,[61], [62], [63]]. Moreover, the CDI coupling and PEG spacer may modulate the AMP immobilization.

The CDI coupling can react with surface -OH groups of the MAO coating, without a catalyst in the reaction. Then, using an appropriate buffer at pH 9.15, the carbonyl of the molecular intermediate becomes available for further binding to the MSI-78 through its free amines [55,56], including its N-terminal group or lysine side chains. Thus, the CDI-based approach promoted a more direct and non-specific (random) peptide linkage.

In contrast, a longer and heterobifunctional HOOC-PEG_113_-MAL spacer was employed for a distinct AMP grafting approach. Using EDC as coupling agent and DMAP as catalyst, the carboxyl from the HOOC-PEG-MAL spacer reacts with the surface -OH groups of the MAO surface. Then, in a proper buffer at pH 6.6, a selective thiol-maleimide conjugation can be promoted [59]. The double bound of the MAL is exposed for subsequent reaction with the thiol (-SH) group of the C-*terminus* cysteine in MSI-78-Cys. This PEG-based approach may potentially enhance peptide's orientation, exposure, and lateral mobility after immobilization, ultimately increasing the surface antibacterial activity [59,63]. To improve even further this potential, we have analyzed two peptide concentrations (0.5 and 1 mg/mL) for the PEG-mediated anchoring approach.

In addition to the covalent grafting strategies, physical AMP adsorption was also tested as a simpler way to immobilize peptides onto the surface. MSI-78 was adsorbed to MAO surfaces, or adsorbed to MAO surfaces previously treated with PEG (*i.e.*, PEGylated MAO surfaces). Thus, the influence of PEG chains on physical AMP adsorption was also studied. It is important to emphasize that, at pH 6.6 (phosphate buffer; Scheme 1B, step IV), the vast majority of amines in MSI-78 are protonated and, therefore, not nucleophilic enough to react with maleimide groups via a Michael Addition [82], favoring physical AMP adsorption.

A common concern with chemical immobilization is potential degradation or alteration of the coating. However, regardless of the AMP immobilization strategy herein used, MAO coatings retained their porous morphology and bioactive composition.

WCA measurements served as preliminary indicators of successful immobilization. CDI-treated surfaces showed increased hydrophobicity, as previously described [56], while PEGylation imparted hydrophilicity due to the polymer's inherent polarity [62]. The further and significant increase in WCA after immersion in peptide solutions suggests AMP immobilization, independently of the strategy adopted for the attachment, attributed to the presence of hydrophobic amino acids (glycine (G), isoleucine (I), phenylalanine (F), leucine (L), alanine (A), and valine (V)) on MSI-78 or MSI-78-Cys. The possible and specific orientation outwards of the hydrophobic N-*terminus* via the more selective conjugation (using the PEG spacer) did not alter this hydrophobic profile of the surfaces.

XPS confirmed surface AMP immobilization through the appearance of nitrogen and bonds that are present in the structure of the molecules. Although N 1s and C 1s spectra could not distinguish between physical adsorption and covalent grafting, the presence of S 2p peaks indicating C–S–C bonding supported successful covalent attachment of MSI-78-Cys to maleimide-functionalized PEG on MAO surfaces.

Elemental N/Ti ratios further elucidated immobilization efficiency. Physically adsorbed AMP (MAO/AMP-0.5) and CDI-grafted AMP (MAO-CDI-AMP-0.5) displayed comparable surface concentrations, suggesting strong adsorption affinity to MAO coatings and/or higher steric hindrance when peptides are directly grafted (via CDI, without spacer). Although the same MSI-78 concentration (0.5 mg/mL) was used to treat MAO-PEG/AMP-0.5 samples, the smallest N/Ti ratio was found for these PEGylated MAO surfaces with physically adsorbed AMP. This result may be attributed to the antifouling properties of PEG against peptide adsorption, decreasing surface AMP concentration. In contrast, MAO-PEG-AMPCys-0.5 and MAO-PEG-AMPCys-1 samples exhibited the highest N/Ti ratios, which may reflect less steric hindrance due to the long PEG chains. Peptide densities obtained indirectly by UV/Vis spectrophotometry aligned with XPS data, with MAO-PEG-AMPCys-1 showing the highest immobilization (2.0 ± 0.5 μg/mm^2^). The retention of AMP after 24 h of incubation under acidic conditions (pH 5.8) further suggested stable immobilization. This condition was chosen because MSI-78 and MSI-78-Cys are more soluble in acidic environments (according to the manufacturer's instructions), potentially increasing their susceptibility to dissolution and detachment from the surfaces. Moreover, a pH of 5.8 more closely reflects the microenvironment of infected implant sites, where local acidification is commonly observed. Supporting this rationale, *S. aureus* can reduce the pH of *in vitro* cultures from 7.2 to approximately 5.6 [83].

This proof-of-concept study aims to identify the most effective AMP immobilization strategy for killing bacteria through direct contact. To simulate clinical conditions, the surfaces were pre-coated with human plasma, which may influence the contact-mediated killing process as it forms a conditioning film composed of hundreds of proteins [84]. Antibacterial assays were performed in PBS (without growth medium) to prevent planktonic bacterial growth, ensuring that bacteria remained in contact with the surface. The incubation period was limited to 5 h, a timeframe that does not affect bacterial viability in PBS. This is also relevant clinically, as the first 6 h post-implantation are critical for bacterial colonization and early biofilm formation [85].

Albumin, the most abundant protein in plasma, is known to reduce bacterial adhesion by occupying binding sites and altering surface energy [33,86]. Martínez Campos et al. [87] reported that albumin readily adsorbs on both titanium and Ca/P-doped MAO coatings, supporting its relevance in the initial stages of implant integration and biofilm prevention. Despite this, viable MRSA were observed on Ti and MAO surfaces. The introduction of CDI (MAO-CDI samples) did not alter bacterial adhesion, echoing previous reports using CDI-treated SAMs [56].

PEGylated Ti surfaces have demonstrated antiadhesive activity against both albumin and bacteria [20,88]; however, these repellent properties were tested separately. Herein, PEGylated MAO surfaces (MAO-PEG) exhibited no significant differences in bacterial adhesion or viability compared to MAO-control. One possible explanation for this result could be a double antifouling effect. Albumin adsorption may be reduced during pre-incubation with human plasma (favoring the subsequent bacterial adhesion). In contrast, the PEG chains may also directly inhibit bacteria colonization. Thus, the total surface adherent MRSA on MAO-PEG may be balanced (and similar to MAO-control).

Compared to MAO-control, all AMP immobilization strategies significantly improved the surface bactericidal activity against MRSA, even under challenging and physiologically relevant conditions that included human plasma proteins and salts (PBS) [89]. Since no significant differences were observed in either N/Ti ratio or AMP density between MAO/AMP-0.5 and MAO-CDI-AMP-0.5 surfaces, it appears that the physical AMP adsorption onto the complex MAO topography provides comparable bactericidal efficacy, in relation to the random and direct covalent grafting by CDI (without PEG spacer). Both strategies may allow sufficient conformational freedom and favorable peptide orientation upon bacterial contact. Furthermore, the similar levels of bacterial adhesion observed for both surface types support a common mechanistic pathway: first, immobilized cationic peptides attract bacteria through interaction with the negatively charged membrane, followed by contact-mediated bactericidal activity [5]. The intrinsic properties of MSI-78 may further enhance this outcome, as its positively charged residues are distributed along the entire peptide chain, facilitating effective electrostatic attraction.

Interestingly, in this work, the more selective AMP grafting using a PEG spacer did not further improve the bactericidal activity, even at higher surface peptide densities (MAO-PEG-AMPCys-0.5 and MAO-PEG-AMPCys-1). This unexpected outcome may be attributed to the structural and physicochemical properties of the PEG spacer chosen (5000 Da). The long and bulky PEG chains may become entangled at the surface, potentially restricting the mobility or proper orientation/conformation of the site-specific grafted peptides. As a result, although bacterial adhesion was still induced by AMP, their subsequent contact-mediated bactericidal action may have been weakened.

The antimicrobial profile was notably enhanced for PEGylated MAO surfaces with physically adsorbed MSI-78 (MAO-PEG/AMP-0.5 samples). The total bacterial adhesion levels were similar to those of the MAO-control, but a significantly greater bactericidal effect was achieved. Even at a lower surface AMP density (0.2 ± 0.1 μg/mm^2^), approximately 80% of adherent bacteria were killed, compared to only 10% on the control, representing a 9-fold increase in killing efficiency. The combination of grafted PEG spacer and low density of adsorbed MSI-78 appears to control the levels of MRSA colonization; consequently, these limited adherent bacteria are killed by contacting AMP present on the porous surface.

SEM analysis revealed pronounced morphological alterations in MRSA, indicating compromised membrane integrity following interaction with AMP-functionalized surfaces, regardless of the immobilization method. Based on mechanisms of action previously proposed for conjugated AMP [5], it is unlikely that surface-immobilized peptides penetrate bacterial cells. Instead, they may exert their effect by destabilizing the membrane's electrostatic balance upon bacterial contact, ultimately leading to cell lysis and death. Future studies are warranted to validate these mechanistic hypotheses, including evaluations over longer incubation periods and against other clinically relevant pathogens implicated in titanium implant-associated infections.

The *in vitro* osteocompatibility of MAO-PEG and MAO-PEG/AMP-0.5 samples was evaluated using osteoblast cultures. Upon implantation, a biological “race for the surface” is immediately initiated, wherein host cells and bacteria compete to colonize the biomaterial [33,88]. Compared to Ti substrates, MAO coatings significantly promoted osteoblast adhesion as early as 4 h post-seeding, potentially reducing the window for bacterial colonization and the risk of early-stage infection. At later stages (up to 15 days), these porous, bioactive surfaces further supported enhanced osteoblast proliferation, suggesting a favorable environment for osseointegration. This excellent behavior is likely attributed to the surface's porosity and the presence of bioactive Ca and P elements, known to play a role in osteogenic response [39,40].

*In vitro* osteogenic behavior was also evaluated using hMSCs (osteoblast precursor cells) cultured under basal and osteogenic conditions. Regarding adhesion and proliferation, we observed that during the early stages of culture (up to 7 days), PEG exhibited its characteristic anti-adhesive effect [90], which was counteracted by the adsorbed AMP. At later time points, the effect of PEG was no longer apparent, likely due to the formation of a conditioning layer of adsorbed biomolecules and cell-secreted extracellular matrix (ECM), resulting in surfaces that were functionally equivalent. Regarding osteogenic behavior, all surfaces supported osteogenic differentiation, as evidenced by increased ALP activity over time (up to 21 days), reaching levels that were up to twofold higher than those of the control group (hMSCs cultured on tissue culture-treated wells).

The antibacterial MAO surfaces with PEG and adsorbed MSI-78 (MAO-PEG/AMP-0.5) did not impair osteoblast or hMSCs viability, adhesion, proliferation, or osteogenic differentiation, underscoring their suitability for bone-related applications. Nonetheless, the exclusive use of *in vitro* models represents a limitation, as these systems lack the biological and mechanical complexity of *in vivo* environments, such as immune cells, dynamic fluid flow, and the full spectrum of biochemical cues present in living tissues. These factors can significantly influence both antibacterial efficacy and osteointegration. Thus, while our findings provide valuable preliminary evidence, *in vivo* studies are required to confirm the long-term biocompatibility and antibacterial efficacy of the AMP-treated MAO surfaces. The MAO technique offers substantial versatility in coating implants of various shapes, as reported in dental implants for mandibular insertion [91] and disk-shaped implants designed for craniotomy procedures [92]. When combined with PEG and AMP post-treatments solutions, MAO-derived surfaces show strong potential for the development of implantable prototypes for preclinical models.

Overall, this study provides valuable insights into different strategies for AMP immobilization onto complex titanium-based surfaces, particularly porous and bioactive MAO coatings. The ability to achieve bactericidal activity, cytocompatibility, and osteogenic behavior makes these surfaces as promising candidates for the development of next-generation of bone implants.

## 5 Conclusion

MSI-78 was successfully immobilized onto MAO TiO_2_ surfaces through physical adsorption and covalent grafting (with and without PEG spacer), providing bactericidal activity against MRSA even in the presence of human plasma proteins (human realistic condition). These different immobilization strategies seem to share the same mechanism of action, first attracting bacteria to the surface, followed by interaction with the membrane to cause its disruption and consequent cell death. Nevertheless, this bacterial attraction may be too high, hindering the effective action of the AMP. Therefore, the best immobilization profile for the complex titanium surfaces was through the combination of grafted PEG spacer molecules together with physically adsorbed MSI-78, resulting in low bacterial adhesion and high killing potency. Importantly, this functionalization approach did not induce cytotoxic effects on bone-like cells and supported osteogenic behavior. These findings highlight the potential of PEGylated MAO coatings with adsorbed AMP for the development of bioactive and antibacterial titanium-based implants, offering a promising solution for reducing implant-associated infections while promoting bone integration.

## CRediT authorship contribution statement

**Natália A. Costa:** Conceptualization, Investigation, Methodology, Validation, Visualization, Writing – original draft. **Cláudia Monteiro:** Conceptualization, Methodology, Supervision, Writing – review & editing. **Liliana Grenho:** Investigation, Methodology, Resources, Validation, Writing – review & editing. **Ana R. Ribeiro:** Methodology, Resources, Writing – review & editing. **Victoria Leiro:** Methodology, Writing – review & editing. **Maria H. Fernandes:** Resources, Writing – review & editing. **Paulo N. Lisboa-Filho:** Conceptualization, Funding acquisition, Methodology, Project administration, Supervision, Validation, Writing – review & editing. **M. Cristina L. Martins:** Conceptualization, Funding acquisition, Methodology, Project administration, Supervision, Validation, Writing – review & editing.

## Declaration of competing interest

The authors declare that they have no known competing financial interests or personal relationships that could have appeared to influence the work reported in this paper.

## Data Availability

Data will be made available on request.
