# Supplementary material for: Exploring immobilization strategies of antimicrobial peptides onto MAO-treated titanium to fight MRSA colonization and preserve osteogenic activity
Source: Mater Today Bio. 2026 Feb 9;37:102896. doi: 10.1016/j.mtbio.2026.102896 (PMC12924753; doi:10.1016/j.mtbio.2026.102896)
Supplement: Multimedia component 1 [file mmc1.docx]

Supplementary data

**Exploring immobilization strategies of antimicrobial peptides onto MAO-treated titanium to fight MRSA colonization and preserve osteogenic activity**

Natália A. Costa^a,b,c,d^, Cláudia Monteiro^b,c^, Liliana Grenho^e,f^, Ana R. Ribeiro^g^, Victoria Leiro^b,c^, Maria H. Fernandes^e,f^, Paulo N. Lisboa-Filho^a*^, M. Cristina L. Martins^b,c,h*^

^a^ UNESP - Universidade Estadual Paulista, Faculdade de Ciências, 17033-360 Bauru, SP, Brazil

^b^ i3S - Instituto de Investigação e Inovação em Saúde, Universidade do Porto, 4200-135 Porto, Portugal

^c^ INEB - Instituto de Engenharia Biomédica, Universidade do Porto, 4200-135 Porto, Portugal

^d^ FEUP - Faculdade de Engenharia, Departamento de Engenharia Mecânica, Universidade do Porto, 4200-465 Porto, Portugal

^e^ BoneLab, Faculdade de Medicina Dentária, Universidade do Porto, Rua Dr. Manuel Pereira da Silva, 4200-393 Porto, Portugal

^f^ LAQV/REQUIMTE, Faculdade de Medicina Dentária, Universidade do Porto, Rua Dr. Manuel Pereira da Silva, 4200-393 Porto, Portugal

^g^ NanoSafety Group, International Iberian Nanotechnology Laboratory, 4715-330 Braga, Portugal

^h^ ICBAS - Instituto de Ciências Biomédicas Abel Salazar, Universidade do Porto, 4050-313 Porto, Portugal

* Corresponding authors.

*E-mail addresses:* cmartins@i3s.up.pt (M. Cristina L. Martins), paulo.lisboa@unesp.br (Paulo N. Lisboa-Filho)


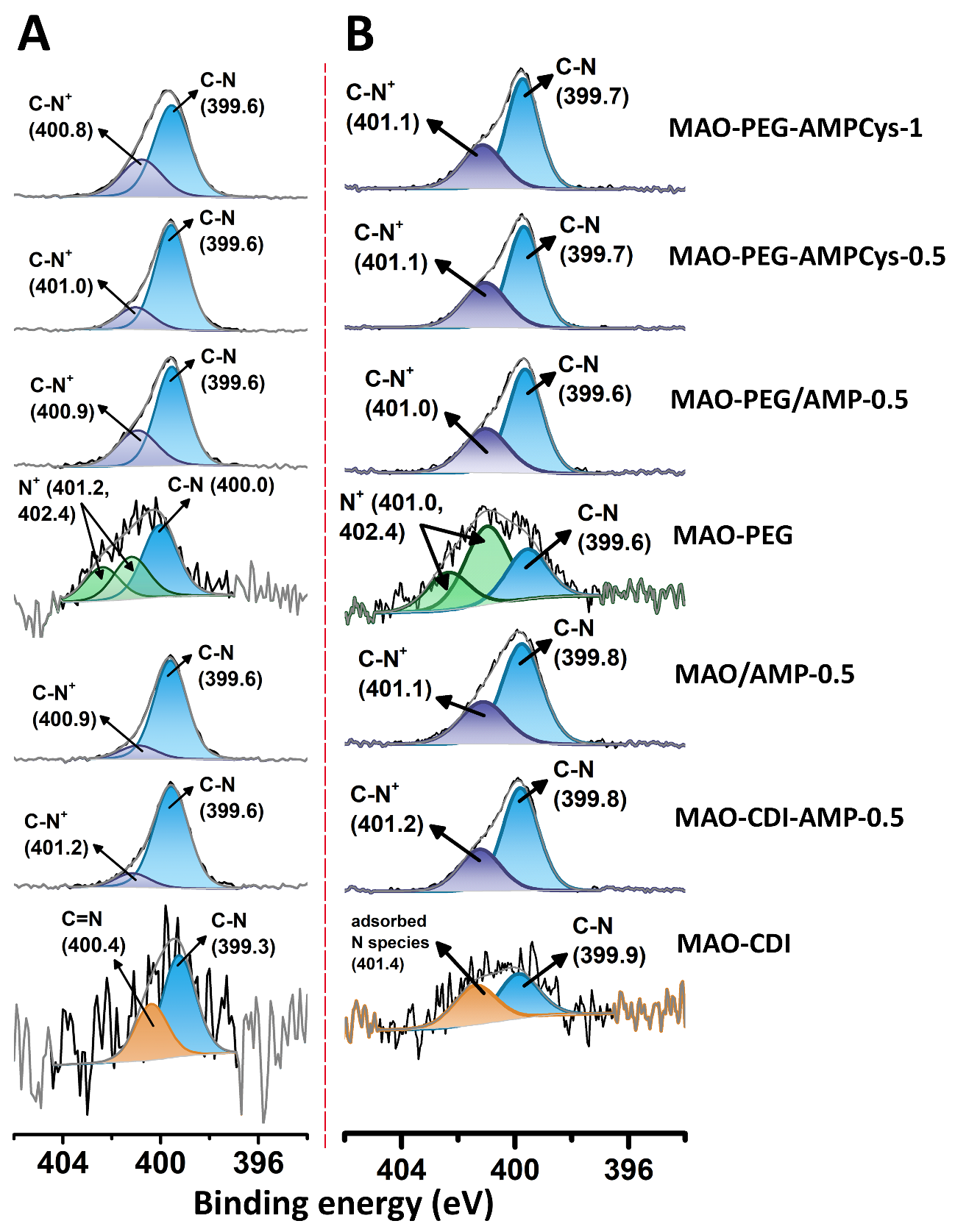


**Fig. S1.** High-resolution XPS spectrum of the N 1s region for the different surfaces, before (A) and after (B) immersion in acidic buffer solution (pH 5.8) for 24 h at 37 °C. Possible contributions are assigned to the fitted peaks (binding energies in eV are given in parentheses). Following incubation, MAO‑CDI surfaces appeared to carry adsorbed nitrogen‑containing species [1].

**References**

[1] S. Bouhadoun, C. Guillard, S. Sorgues, A. Hérissan, C. Colbeau-Justin, F. Dapozze, A. Habert, V. Maurel, N. Herlin-Boime, Laser synthesized TiO2-based nanoparticles and their efficiency in the photocatalytic degradation of linear carboxylic acids, Sci. Technol. Adv. Mater. 18 (2017) 805–815. https://doi.org/10.1080/14686996.2017.1379858.
